# “A good collaboration is based on unique contributions from each side”: assessing the dynamics of collaboration in stem cell science

**DOI:** 10.1186/s40504-017-0053-y

**Published:** 2017-05-04

**Authors:** Michael Morrison

**Affiliations:** Centre for Health, Law and Emerging Technologies (HeLEX), Nuffield Department of Population Health, University of Oxford, Ewert House, Ewert Place, Banbury Road, Oxford, OX2 7DD UK

**Keywords:** Collaboration, Stem cells, Public-private partnership, Moral economy

## Abstract

The rise of ‘big biology’ is bringing academic and industrial scientists together in large consortia to address translational challenges in the life sciences. In order to assess the impact of this change, this paper examines the existing norms and styles of collaboration in one high profile translational domain; stem cell research. Data is drawn from qualitative interviews with academic and industry scientists working in a large European stem cell research project. Respondents discussed what they perceived as the main benefits and risks of collaborative research, what styles of collaboration they were familiar with, and what collaborative work in stem cell science normally involves. A wide range of materials, data, and expertise can be exchanged during collaborative work. Informal collaborations are governed by an ethos of reciprocity and mediated by trust while formal project agreements can provide a safe space for sharing between unfamiliar partners. These characteristics make stem cell research well suited to pre-competitive public-private ventures but translation of new products to market may be more challenging.

**Electronic supplementary material:**

The online version of this article (doi:10.1186/s40504-017-0053-y) contains supplementary material, which is available to authorized users.

## Introduction

Large-scale public-private consortia are increasingly being employed to facilitate translational research in the life sciences (Lezaun 2013). Contemporary examples include the Quebec Consortium for Drug Discovery (CQDM), several networks created under the ‘Investments for the Future’ programme of the French National Research Agency (ANR), and the Innovative Medicine’s Initiative (IMI), a joint venture between the European Commission and the European federation of pharmaceutical industries and associations (Efpia). Public-private collaboration is seen as especially relevant for addressing the translational challenges of stem cell science and regenerative medicine (Rao 2013; Bubela et al. 2014; French et al. 2014). There are currently two IMI consortia in the stem cell field; Stem cells for Biological Assays of Novel drugs and prediCtive toxiCology (StemBANCC) and the European Bank for induced pluripotent Stem Cells (EBiSC). Both projects bring together multiple European universities and pharmaceutical companies to collaborate on developing stem cell technologies as a platform for drug discovery. An outline of one of these projects is sufficient to confer a sense of their scale and scope. One of the larger IMI consortia, StemBANCC involves 35 partner institutions spread across ten countries. Twelve consortia members are pharmaceutical or biotechnology companies. As with several other instances of ‘big biology’, a key aim of both IMI stem cell projects is to facilitate “greater and faster circulation of informational and biological materials” (Lezaun 2013, p480). In the case of StemBANCC this means production, characterisation, and dissemination of up to 1,500 induced pluripotent stem cell lines over 5 years.

Neither scientific collaboration (Vermeulen et al. 2013) nor academic-industry interactions (Nordlund 2015) are novel or uncommon in the life sciences. Stem cell research in particular is characterised by a high degree of international alliances, networks and collaborations (Isasi 2012; Luo and Matthews 2013). However, despite cases like the European Molecular Biology Organisation, large-scale consortia-style collaborations have not traditionally been the norm in the life sciences (Davies et al. 2013; Vermeulen et al. 2013). Unlike fields such as high-energy physics where mass collaboration has been “spectacularly institutionalised” (Cronin 2001, p.564), the rise of so-called ‘big biology’ is a relatively recent occurrence, following the successful example set by the Human Genome Project (Davies et al. 2013). Historically, innovative or unprecedented types of interaction between academics and commercial partners have proven disruptive. When Axel Westman became one of the first academic physicians in Sweden to co-develop drugs with an industry partner in the 1930s (Nordlund 2015) or when US cell biologist Leonard Hayflick first provided human cell cultures to pharmaceutical companies on a for-profit basis in the 1970s (Hall 2003), their actions were counter to prevailing local norms of conduct in the life sciences at the time. These actions would be considered unremarkable today but each generated criticism and opprobrium (especially in Hayflick’s case) from the scientific and medical establishments when they occurred.

In addition, major translational ventures have been characterised as “multi-directional and multi-modal” enterprises where “objects, knowledge, practices and resources are circulated between multiple sites” (Lewis et al. 2014). These flows are not unproblematic; they require work to cross the boundaries, for example between institutions, disciplines, or sectors. As Lezaun (2013) notes large collaborative ventures actually require scientific materials and data to be *devalued* by the scientists who produce them, in order to make them freely shareable with others who might, in other circumstances, be competitors. These factors, especially taken together, raise the question of whether the existing norms, patterns and traditions of collaboration in various life sciences fields are likely to support or hinder working in large-scale public-private consortia. The question is particularly pertinent as the public-private model of ‘big biology’ is becoming more common (for example more than 70 IMI consortia have so far been created, with more in the pipeline).

This paper aims to contribute to the burgeoning social science literature on ‘big biology’ (Calvert 2010; Davies et al. 2013; Lezaun 2013; Vermeulen et al. 2013) by exploring the potential impact of large public-private consortia in the field of stem cell research. This will involve drawing on data from a series of qualitative interviews conducted with stem cell scientists working on the IMI StemBANCC project. That project, a multi-institutional, international public-private consortium, exemplifies the novel translational infrastructures becoming increasingly prevalent in the life sciences. The aim of interviews was not to elicit respondents’ views on StemBANCC, but to explore their previous experiences of collaborative working. This is because the consortia project was ongoing at the time of interviewing and respondents’ impressions of it were likely to still be in the process of formation. Their prior experiences provide a better route to drawing out and understanding the *existing* norms, motivations, and expectations of collaborative work in stem cell science. It is these existing ‘unwritten rules’ about what counts as good or appropriate scientific behaviour – what Daston (1995) and others have described as the moral economy of science – that this paper is interested in exploring, because it is these prior norms that may support, or be challenged by, novel collaborative enterprises.^1^ As a public-private consortium, StemBANCC includes scientists working in academic institutions and those in large pharmaceutical firms. This allowed the findings to include perspectives from both academic and industry scientists, something often neglected in previous research, even where university-industry collaboration has been the topic of study (Ankrah et al. 2013).

One of the traditional limitations of the qualitative research is that it involves small samples sizes with low generalisability. In social research this is generally accepted as a worth-while trade-off in return for more detailed accounts of participant’s behaviour. This is also the case with the research reported here, where the sample size n = 16. The broader topic of scientific collaboration is the subject of a large and diverse academic literature. This includes contributions from history, philosophy and sociology of science, information science, organisational studies, and research policy (Sonnenwald 2007). There are many quantitative and Scientometric studies of collaboration as well as qualitative and theoretical approaches. To off-set the small sample size, wherever possible findings from interview data will be contextualised by reference to general trends or patterns detected by these larger studies. This paper will proceed by expanding on the concept of scientific collaboration, providing a brief overview of the main forms, drivers, and challenges of collaboration drawn from the wider academic literature. This will introduce the key parameters that will be used to evaluate and contextualise respondents’ accounts of collaboration in stem cell science. The notion of moral economies of science will also be elaborated upon and its relevance to studying and understanding collaboration set out. The analysis of stem cell scientists’ discourse will identify commonplace organisational forms, trends and formal or informal rules of collaboration in that field. In particular attention will be given to aspects that appear likely to support or challenge current and future use of large public-private consortia in stem cell research. Following this assessment, future issues for governance and policy attention will be highlighted.

## Theorising collaboration

### Defining scientific collaboration

‘Scientific collaboration’ can potentially describe a diverse array of practices. Two scientists working on a particular experiment, a multinational clinical trial producing papers with dozens of authors, or out-licensing a patented discovery through a university technology transfer office could all be considered examples of collaborative work (Katz and Martin 1997). To keep the analysis manageable it is necessary in this paper to adopt a narrower definition of collaboration. Hackett (2005b, p.788) regards the research group as “an elemental form of scientific collaboration and knowledge production”, at least within the life sciences. Members of a research group work together to prepare materials, design and carry out experiments, and write papers. However, as Shrum et al. (2001, p687) argue “[i]n modern scientific collaborations, the relationships of interest are often between structural components such as teams”. In keeping with this insight, the type of collaboration of most interest here is scientific activity that takes place between two or more research groups. This includes collaboration between different academic research groups and between academic research groups and scientists working in industry.

### Drivers of collaboration

A number of different elements have been identified that foster scientific collaboration. These can roughly be divided into internal and external drivers. Internal drivers describe the motivations of individual scientists that lead them to seek out collaborative working arrangements. As scientific skill sets become increasingly specialised, collaboration with others offers a means to address bigger, potentially more financially and intellectually rewarding research questions (Katz and Martin 1997; Hampton and Parker 2011), and as a way to produce more, and more highly cited publications (Luo and Matthews 2013). Scientists must therefore make strategic decisions about who to collaborate with in order to realise the most productive outcomes (Atkinson et al. 1998). Leahey and Reikowsky (2008) further develop the typology of collaboration, distinguishing between complementary and reinforcing styles of collaborative work. ‘Complementary’ arrangements occur when each collaborating partner (or research group) brings a specific skill set and makes a unique contribution to the work based on a clear division of labour, whereas ‘reinforcing’ collaborators have overlapping skills sets and tend to routinely work together on shared projects. They also identified mentoring arrangements where established scientists work with junior colleagues and ‘novelty generating’ collaborations where researchers seek out new collaborators to investigate a new topic area as important patterns of scientific activity. All the above models of collaboration are at least potentially feasible in stem cell science.

External drivers of collaboration are exogenous factors that put pressure on scientists to work together. In an academic environment that is increasingly competitive and subject to bureaucratic oversight and management, collaboration offers a way to access resources and funding (Hackett 1990). The demand, especially for funding, drives collaboration with other academics and industrial partners:

“studies have shown that researchers manage very well to navigate changing institutional conditions and shape them to their advantage, using industry collaboration as an asset for gaining experience, opening new research opportunities, and not least advertising to funders and other stakeholders their activities as being socially or economically relevant” (Hallonsten 2014, p.354).

From an industry perspective, collaborations with academic scientists are regarded as a way to increase firms’ capacity to innovate and access new markets in an era of rapid technological change and global competition by providing access to additional resources, knowledge, and technologies (Morandi 2013; Ankrah and AL-Tabbaa 2015). Funding specific projects is also a way for states and other actors to promote scientific collaboration in strategic areas. This is exemplified by the European Union’s framework programmes, which were designed to foster cross-border collaborative research in key areas of science and technology (Gusmão 2001). These accounts of academia and industry both fit what Hallonsten (2014) describes as the ‘resource dependence’ model of scientific collaboration. This also suggests a practical definition of scientific collaboration as an activity in which resources, of one kind or another, are exchanged, or shared, between collaborating research groups.

### Governing tensions in scientific collaborations

Scientists working in similar areas are generally in competition for publications, prestige, external grant funding, and institutional positions. Collaboration does not necessarily mean the absence of competition. Rather collaboration and competition can co-exist, with scientists and research teams in working in collaboration also capable of competing with one another at the same time (Atkinson et al. 1998). This can create potential for conflict, especially between research teams, which can actually be exacerbated in larger, more multi-institutional projects:

As scientific projects encompass multiple organisations, the potential for conflict grows because its sources are embedded in the interaction system: functional differentiation between subunits, staff heterogeneity, divisions between scientists and project managers, styles of supervision, forms of power and reward systems. Conflicts may be caused by competition for resources, dissatisfaction over the discharge of tasks and claims for credit […] as well as by differences in technical approaches and scientific interpretations (Shrum et al. 2001, p684).

There is no single, universal set of organisational arrangements for managing scientific collaborations and assuaging conflicts. Chompalov et al. (2002, cited in Sonnenwald 2007, p.659) identified a spectrum of collaborative arrangements among physicists from ‘bureaucratic collaborations’ with a “hierarchy of authority, written rules and regulations, formalized responsibilities, and a specialized division of labour” to ‘participatory collaborations’ with “no formal rules and regulations but rather nonbinding memos of understanding” (Sonnenwald 2007, p.660). Each of these are possible collaborative models for academic stem cell science.

Bureaucratic management of science by universities and funding agencies can impose external, formal rules on scientists (Hackett 1990; Braun 1998). Increased competition for funding and tenured positions gives institutions greater scope to impose performance requirements such as annual targets for generating high-status publications, intellectual property, or grant income. Similarly funding agencies have considerable leeway to impose administrative and financial requirements on academic scientists in return for economic support (Braun 1998). EU projects, for example, tend to come with a very specific set of bureaucratic management requirements, in terms of funding regulations, mandatory reporting on progress, pre-agreed milestones and deliverables et cetera. In the absence of such formal arrangements, the, the idea of trust, both in terms of being able to trust a collaborator’s motives, and in terms of having confidence in their scientific competence, has been understood to be a key factor in allowing collaborations to work (Shrum et al. 2001). Trust, or at least ‘trustworthy conduct’, can be regarded as part of a moral economy of science (Daston 1995; Nordlund 2015).

Moral economies are normative in that they “define and maintain scientific ideals” (Nordlund 2015, p.50). Unlike the previous Mertonian ideal of scientific norms, moral economies are not abstract and universal but anchored in practices such as accurate measurement and variable over time and place (Daston 1995). This is illustrated by the director of a laboratory working on model organisms, cited in Hackett (2005b, p.809), who explained “Once you know enough about it that it seems to be time to publish something, then the appropriate thing to do is to share what you’ve got so whatever you’ve reported can be reproduced”. This comment describes the normative obligation in this particular field of research that, once a study is published, the research group responsible is expected to make the study data and materials available to other researchers. However, the same researcher went on to describe the strategy of presenting *unpublished* data in the form of a talk, allowing the research group to stake a claim to priority over a particular discovery while buying more time to keep their materials and data exclusive while they conducted further experiments (Ibid). This shows how the requirement of a moral economy to share data can be sufficiently flexible to manage the fact that colleagues in the same research field are also likely to be competitors for publications and the resources that scientific success brings.

The situation is different for industry collaborations. For life sciences companies, scientific knowledge is typically not shared because doing so would undermine a firm’s ability to commercialise products based on it. This creates a potential tension in academic-industry collaborations between academics desire to (strategically) release data and materials and companies’ need for longer-term secrecy to ensure they can exploit the fruits of their investment (Evans 2010). This tension is sometimes summarised in the shorthand notion of ‘patents versus papers’ although the balance of risks and incentives for all parties in university-industry collaboration is more nuanced and more complex than this would suggest (Morandi 2013; Ankrah and AL-Tabbaa 2015). Moreover universities are increasingly invested in managing their own intellectual property rights and have their own interests in controlling the release of information. Evans (2010) argues that it is not that academic scientists do not keep secrets, but rather than they are not as strict at keeping secrets as industry partners would sometimes like them to be. As a result when academic scientists collaborate with industry partners it is almost always a formal, contractually managed project with a clear division of labour and written rules and regulations covering the disclosure and dissemination of any new knowledge or material produced during the course of the collaboration

Having described these basic parameters of scientific collaboration, the next step is to consider in more detail the empirical case study.

## Case study and methods

### The StemBANCC consortium

The StemBANCC consortium comprises 22 public sector institutions (universities and state-supported research institutes) and 12 industry partners (large pharmaceutical companies or small to medium enterprises) plus a for-profit project management firm. The large number of partners and involvement of multiple industry organisations in the same collaboration are unusual for the life sciences, but exemplify the large-scale translational arrangements promoted through the Innovative Medicines Initiative. The IMI builds on existing European funding mechanisms; IMI consortia created 2008–2013 (designated ‘phase 1’) are funded through the EC Seventh Framework Programme (FP7), while projects initiated 2014–2020 (‘phase 2’) operate through the Horizon 2020 programme. Public sector partners are invited to put together bids to work on pre-defined topics. A selected number of viable bids are then invited to negotiate with industry partners, with a final successful public-private consortium being awarded the funding. There are several additional arrangements to facilitate the public-private nature of IMI consortia. Funding is provided to the public sector partners and small to medium enterprises in each consortia. The Efpia (pharmaceutical industry) partners in an IMI consortium do not receive direct financial support. Instead they provide resources and staff time approximately equivalent in value to the public funding allocated to each specific IMI project. StemBANCC’s total €55.6 million budget entails €26 m from the EC, a €21.5 m Efpia ‘in kind’ contribution and an additional €8 m of ‘other’ financing. Each IMI consortia is jointly managed by a public and private sector institution.

In drafting, enforcing and arbitrating agreements between the public and private partner institutions in these consortia the EC acts as a ‘neutral broker’ (Goldman 2011). A project agreement, drafted and signed by all partners before any work commences, sets out the terms of the collaboration including the intellectual property rights of each partner. Perhaps the most significant aspect of the IMI terms is that the research is considered to operate in a ‘pre-competitive space’ (Bubela et al. 2014). IMI consortia are intended to address ‘upstream’ issues in the drug discovery process. The goal of StemBANCC is to produce 1,500 induced pluripotent stem cell (iPSC) lines from patients with a range of common chronic conditions and healthy volunteers and to develop cell-based assays for toxicological use. There is a significant expectation that using human stem cells for toxicology and pharmacology testing could provide more accurate results than currently employed animal models and eliminate unsuitable drug candidates in preclinical investigation. The (future) availability of these iPSC is intended to accelerate the development of stem cells as a platform technology for assessing the toxicology and biological activity of small molecule drug compounds (Author 2017). This aligns with Lezaun’s observation that ‘big biology’ projects often have an orientation towards producing ‘user friendly’ tools and resources that, at the same time, carry implicit assumptions about what future research is worth doing. The public-private consortium format was employed because this translational task is considered to require the combined skill sets of both industry and academic sectors, and to be in the near-to-medium term interests of both groups. A further component of the public-private settlement is that the 1,500 iPSC created will be considered a communal resource, made available to the wider academic and industry stem cell research communities by the end of the project. The majority of materials and data produced by these consortia are thus not capable of being patented or subject to trade secrets by any of the partner institutions during or after the research.

### Study method and data analysis

StemBANCC commenced in October 2012. The author was employed on the project to help secure research ethics committee approval for recruitment of tissue donors and contribute to internal project governance mechanisms (see Author et al. 2015). Through this involvement it was possible to identify suitable academic and industry scientists employed on StemBANCC with experience of working with stem cells. In keeping with the heterogeneous nature of stem cell science, these included cell and molecular biologists, geneticists, developmental biologists, pharmacologists, toxicologists, and others who employ cell cultures as tools to evaluate pharmacologically active compounds. Some scientists were based in a clinical setting, e.g. neurology or endocrinology, some were primarily laboratory-based ‘bench’ scientists, and others operated in both contexts. An interview schedule was devised, based on a review of the literature outlined above. Questions were designed to elicit respondents’ experiences, expectations and attitudes to research collaboration (The questionnaire is provided in Additional file 1). Sixteen stem cell scientists responded to the invitation to take part in a qualitative study. Research ethics approval was obtained for this work as listed at the end of the manuscript (separate from the main StemBANCC study). Interviews were carried out between March and August 2015, face to face or using digital video communication software. The latter option was used to interview geographically dispersed StemBANCC participants. Written informed consent was collected from all respondents before each interview.

Interviews were transcribed and analysed using the NVivo 10 software programme. An integrated approach incorporating both deductive and inductive coding strategies was adopted (Bradley et al. 2007). The interview schedule provided the key conceptual domains of a deductive coding framework; the extent of respondents’ prior collaborative experience, types of previous collaboration, norms concerning what (if anything) was commonly shared with collaborating research groups, what respondents perceived as the main benefits and risks of collaboration, and attitudes to collaborating with academic or industry partners. Transcript text collected under these broad headings was then reread and a second round of inductive sub-codes developed in response to new and emerging themes in the data (Corbin and Strauss 1990). Interviewees were anonymized to encourage them to speak more freely. Quotes from participants are identified in the subsequent text by the following scheme: ‘EAS’ indicates an established academic scientist, ‘EIS’ indicates an established industry scientist, ‘ECAS’ indicates an early career academic scientist and ‘ECIS’ an early career industry scientist. Each individual participant is identified by one of these letter codes plus a number specific to that participant; e.g. ‘EAS 5’ denotes ‘established academic scientists 5’. Respondents were classified as ‘academic’ or ‘industry’ scientists on the basis of their affiliation at the time of interview – i.e. whether they were working for a public or private sector institution involved in StemBANCC. Researchers were classified as ‘early career’ if they were in a PhD student or postdoctoral position for academics, or in their first industrial job. The details of many consortium members are listed, along with photographs and professional affiliations on the project website meaning that even basic conventional attributes such as gender could be potentially identifying if taken in combination with the information already disclosed.

### Characteristics of the respondent group

The respondent group was biased towards established academic scientists, although this does reflect the greater number of academic institutions in the consortium. (Table 1).

**Table 1:** Breakdown of interviewees by sector and career stage

	Established researchers	Early career researchers
Public sector (academic)	9	3
Private sector (industry)	3	1

All of the academic scientists interviewed had previous experience of collaborating with other academics. Most of the established academic scientists had at least some prior experience of working with industry although this was usually reported as being less extensive that their prior collaborations with other academics. For the early career academic researchers StemBANCC was more likely to be their first experience of conducting research with industrial partners. All of the industry scientists had previous experience of working with other research groups within the same company and of carrying out collaborative research with scientists in academic institutions. All the interviewees thus had some experience which they defined as ‘collaborative’ to draw on. The familiarity of most of the academic and industry scientists with academic-industry collaboration also reflects the fact that academic-industry relations are both a long-standing phenomenon and one which has become increasingly common in recent decades (Ankrah and AL-Tabbaa 2015).

## Results

### Resource exchange in collaborative science

Respondents described a range of things which might be made available to partners in the course of a collaboration. These included data (for example readings produced by various instruments during the course of an experiment), protocols and methods, research objects and tools (including cultured cell lines), tacit knowledge, expertise and advice, and even people (primarily PhD students and postdoctoral researchers who might temporarily join a laboratory to learn new skills before returning to their original research group). This expands on the ‘resource dependence’ model of collaboration to include a wide range of useful components – tools, material, data, expertise, access to specialised equipment et cetera–that might be brought ‘inside’ the research group from an external partner. In return some element of the group’s know-how and/or material resources may be made accessible to those normally ‘outside’ the group:

“a good collaboration is based on unique contributions from each side so that we can share things that we don't… and gain access to things that we don't have, the techniques and know-how and reagents” EAS 2

“usually it is when we can see a clear benefit for building up expertise, getting access to certain cell models or animal models or any particular know-how that we don’t have internally, so that’s usually the driver” EIS 4

Respondents identified the benefits of collaboration in terms of being able to address more complex research questions, increasing efficiency in getting scientific work done, and for academic partners, increasing publication output; “we end up on their papers, they end up on our papers” EAS1. Our operational definition of ‘research collaboration’ can therefore be refined to include any interaction between scientists through which two or more research groups are brought into alignment, allowing them to combine resources and so increase the scope of ‘do-able’ scientific work (after Fujimura 1987).

More ambitious research projects in induced pluripotent stem cell science were made more do-able, not only by an increase in overall resources (materials and people), but through a particular division of labour:

“you need the clinical team who understand the disease and have access to the patients in the first place, you need our skill set for working with iPS cells, you need another skill set for looking at the differentiated cells, and the electrophysiologists if it’s a neuronal project, and all of that” EAS 7

This suggests a complementary (rather than reinforcing) style of inter-group collaborative working based on disciplinary or sub-disciplinary skill sets and expertise. Respondents working on cell culture and/or cellular reprogramming mentioned ‘analytics and data management’, ‘electrophysiology’, and ‘establishing clinical relevance’ among the types of tasks to be outsourced to ‘other’ groups such as bioinformaticians, neuroscientists, or clinicians during collaborations. Stem cell science, like regenerative medicine, is a heterogeneous (Zhao and Strotmann 2011) and interdisciplinary (Calvert 2010) field. It requires intellectual and practical contributions from scientists with a variety of training and skill sets including cell and developmental biologists, molecular biologists and geneticists, clinicians, bioinformaticians, and others. The above accounts present a model of complementary collaboration, with a modular, task-based organisation of life sciences research similar to that depicted by Fujimura (1987). They also appear to support the claim made by Centellas et al. (2014) that cross-disciplinary collaborations can actually act to reinforce distinct disciplinary identities and roles rather than ‘blurring’ these boundaries, although it should be noted that within StemBANCC several scientists held joint clinical and laboratory interests that also fit Calvert’s (2010) individual model of interdisciplinarity.

The need to tap additional skill sets in complex areas like stem cell research is also an important factor in promoting collaboration for industry scientists. The industry scientists interviewed reported another way in which the *nature* of the task(s) to be outsourced has an influence on the choice of collaborative partner:

“So, collaborating with external academic partners, in the way that we do through StemBANCC, basically allows us to access innovations that we wouldn’t necessarily develop ourselves […] If you begin to think, rather than an academic collaboration, about a collaboration with a CRO, that becomes more like, again accessing skills that you don’t necessarily have in-house, so for example I outsource a fair amount of behavioural pharmacology, to support projects because we don’t have a behavioural pharmacology team here, or indeed an electrophysiology team, so I outsource those to, those experiments to CROs. And there it’s more like ‘another pair of hands’” EIS 3.

This adds an additional dimension to the drivers of collaboration from an industry perspective; there is still a modular, skill and task-based division of labour, but standardised (or ‘packaged’) tasks are more likely to be outsourced to Contract Research Organisations (CROs) while more ‘exploratory’ open-ended or innovative research tasks tend to be allocated to academic collaborative partners. These findings suggest that collaborations in stem cell science arise as both a means to counter the limitations of increasing scientific specialisation and the need to access a variety of resources, from capital to data from external sources (Hallonsten 2014; Leahey and Reikowsky 2008). This gives some insight into the benefits and drivers of collaboration but it does not yet reveal a great deal about the values in play in collaborative working.

### Moral economies in collaboration

Echoing Katz and Martin (1997) there was no single element which was common to all research collaborations. However, much of what scientists were prepared to share with collaborators had not (yet) been made available to the wider scientific community through publication in a scientific journal, or embedded in a patent, commercially available product, or service. Indeed, one reason given for collaboration was to get access to unreleased knowledge “because everything else is available to everyone freely” (EAS 2). Collaborations involving the sharing of unreleased material tended to be characterised by an ethos of reciprocity:

“I'm always sharing unpublished data is the answer. And, even more so with collaborators. I mean what winds me up about, you know, people that I've stopped collaborating with are actually the ones where I think, you know, if they're not happy to see it as a two-way interactive thing, where we're both sharing data. I'm just not interested in the long term, I can't be bothered, there's no point” EAS 4

“[O]ne important part of a collaboration is that that everybody gets some benefit out of the whole process” EIS 2

Sharing unpublished data, as the first quote makes clear, is expected to be a ‘two way’ process, with each collaborating partner being open to exchanging resources held by their research group with the others in the collaboration. Experimental data, as products of a research group’s labour and its distinctive ensemble of technologies, forms the basis for the outputs on which the group is valued, whether this is publications and talks or patents, trade secrets and products (Hackett 2005b; Evans 2010). Experimental results also open up new avenues of enquiry that will inform the future work of individual scientists and research groups. This is work whose value has not yet been realised and is therefore not to be given away, or given up lightly. Indeed, the thing that respondents most frequently cited as a risk associated with collaborative research was the possibility of unequal reciprocity from a partner. Paralleling the twin issues of a collaborator’s motives and competence described by Shrum et al. (2001) this included both malicious action, such as ‘scooping’ research ideas or data by failing to give due credit to collaborating partners, but also poor scientific practice resulting in a partner providing late, irrelevant or low-quality data.

As with previous studies (Grubb and Easterbrook 2011), the number of respondents who mentioned being scooped as a concern was higher than those who recounted actual prior experience of being scooped. While being scooped is an unpleasant and irritating experience for a senior academic, it is potentially more damaging, and thus a particular worry, for more junior scientists:

“[A]s an academic I have a non-permanent contract. Part of my kind of score sheet that gets me the next contract is things like publications so if you have a collaboration whereby, […] someone kind of publishes without you or takes your kind of intellectual input and shares it with other parties that then removes the novelty of your work, that’s a big thing because, you know, if you spent however long and invested however much and then all of a sudden you’re not impacting in a way which will affect your career” ECAS 1

This is an important reminder that collaborations are not only ‘horizontal’ arrangements between established specialists but also involve vertical collaborative arrangements between established and more junior scientists (c.f. Leahey and Reikowsky 2008, p.435). Research groups are ‘filter feeders’ to which graduate students and early career researchers contribute while simultaneously trying to leverage the value of this work to “escape the orbit of the lab head and establish independent careers” (Hackett 2005b, p793). Early career researchers are more vulnerable to setbacks, such as being scooped, as they typically have less reputation and job security to fall back on if something does go wrong. Protecting the interests of junior members of a research team when establishing a collaborative venture can be one way in which lab heads and senior researchers demonstrate their care of the group (Davies and Horst 2015) as in the following example:

“[I]f I have a Post-Doc or a PhD student working on such a project, obviously I guess I tend to try and be a little bit protective of that particular project, which wouldn't stop me from any collaboration, but this is always something we bring up very openly” EAS 8

These examples demonstrate that while collaboration may be driven by strategic requirements and the need to access resources, it is still governed by a normative regime of collegial responsibility, characterised by a responsibility to share with those who share with you and to pay attention to the interests of junior researchers. These norms are not, of course, absolute, as incidences of bad faith do occur.

### Styles of collaboration; Formal and informal collaborations

With a view to stimulating discussion about different degrees of hierarchical and bureaucratic management in research collaboration, a distinction was proposed by the interviewer between ‘formal’ collaborations involving ‘formal agreements, deliverables and milestones’ and informal or less formal collaborative ventures. Examples of prior work identified by respondents as ‘formal’ included projects organised through other EU-funded schemes including Horizon 2020 and the Framework programmes, and other consortia supported by a range of organisational types including state funding, charities and pharmaceutical companies. EU projects in particular were used to describe the components that marked a collaboration as formal:

“within official collaboration projects for example, funded by the European Commission or by [national funding organisation], we have agreements similar to that used in the IMI project, so the collaboration and the exchange of confidential data and of IP issues is regulated and approved by all partners” EAS 5

For scientists working in the industrial sector, the meaning of, and requirement for, formal projects was relatively straightforward and ubiquitous:

“[I]f we have a collaboration outside the company it’s always, or it should be formal in some way, some agreement that defines the collaboration” EIS 4

“[W]e have […] projects that runs into the core business and those, we don’t share anything unless we have strict agreements, or the more sort of academic endeavours that we also have ongoing here, we share unpublished data within these collaborations” ECIS 1

The importance of formal, legal agreements between companies and external agents reflects firms’ traditional reliance on secrecy and restricted dissemination to secure control and profit from their innovations. A distinction was also made between ‘core’ internal knowledge and data, which is most tightly guarded (at least until it can be secured as a product or patent) and data generated with, and for, academic collaborators, which is intended for at least some level of dissemination. On the academic side, the prevalence of formal collaborative arrangements reflects the dominance of the fixed-term project as the *de facto* organisational unit of contemporary scientific research, and the dependence of scientists (and academic institutions) on external funding (Hackett 1990; Shrum et al. 2001). Academic scientists also recognised the desire to employ formal legal agreements governing ownership of the outputs of scientific collaborations was fostered by universities, and in particular academic technology transfer offices, as well as coming from external sources such as funding agencies or industry partners.

Formal projects, underpinned by contractual agreements governing what can or cannot be done with collaboratively generated materials and data can provide a ‘safe space’ for both academic and industrial scientists to share unreleased data, but also know-how and tacit knowledge which is not generally incorporated into publications or patent applications:

“In the European projects, this is really basically everything. So we share knowledge, data, protocols in an extremely open system, because we're all working on the same team and it's very important that people are doing the best quality research” EAS 6

“[W]ithin our work package [in the IMI consortium] we are very open, as in we will share protocols down to reagent numbers, down to catalogue codes and throughout the day we will help troubleshoot each other’s experiments” ECAS 1

At the same time, some academic respondents also felt project agreements and legal arrangements made collaboration more complex and created an additional bureaucratic workload that could delay scientific work. The bureaucratic burden of collaboration was seen as particularly onerous when commercial partners were involved. This was reflected in the talk of scientists interviewed for this research; discussions of industry collaborations abound with acronyms such as MTA (Material Transfer Agreements), NDA (Non-Disclosure Agreement) and CDA (Confidential Disclosure Agreement), which refer to the kinds of formal contractual agreements that govern particular types of academic-industry collaborative working. A few academic respondents felt that industrial partners tended to be more reticent when it came to sharing data and materials, while some industry respondents felt that on occasion academic scientists were potentially liable to inadvertently disclose potentially valuable information (c.f. Evans 2010). Some of the discussion from both academic and industry scientists about academic-industry collaborations thus reflected the common ‘publications versus patents’ model of understanding divergent academic and commercial interests. However, one industry scientist offered a more nuanced take on underlying reason for potential tensions in academic-industry collaboration:

“I sometimes feel that there is a language gap between academics and industrial scientists and I think that’s partly due to the fact that, I think, academics publish everything so it’s very visible what it is that they are doing and what their research interests are. I think that the drug discovery process and what we’re doing in industry may be rather opaque to the academic population who don’t have first-hand experience of the process and don’t understand how the process hangs together and the sorts of data that we want at a specific point” EIS 3

This suggests that the kinds of calibration and (re)alignment work that Lewis et al. (2014) described in relation to research spanning the laboratory and the clinic is also involved to bridge the worlds of the university and industry laboratories. It should be noted that neither academic nor industry scientists interviewed presented a homogenous account of academic-industry collaboration as challenging or difficult; rather it was something whose quality varied according the partners and the nature of the collaboration. There were clearly benefits and challenges to taking part in formal collaborations, something which in any case was largely unavoidable given the contemporary landscape of science funding and innovation.

If formal collaborative research was defined in terms of externally imposed financial, legal and organisational requirements, then ‘informal’ collaborations were largely identified by the absence of one or more of these elements. In the following example ‘informality’ is marked by the absence of an explicit legal or financial contract between parties:

“[S]ome of these things can be organic as well, they don't have to be necessarily to do with funding. I could have a question that an expert knows, or I could have published something and somebody wants my cells, or something like that” EAS 6.

In these cases, where the full range of safeguards provided by a ‘formal’ project agreement are not in place, careful weighing of the risks and benefits of releasing unpublished data to a particular collaborator must be made:

“[T]he cardiologist we deal with in [name of institution] we’ve known him for many years, he used to be professor of cardiology here […] so you know he is a trusted confidant, but if we’d just met someone at a conference who came up to us […] then we would think twice about, especially if something had a good chance of getting into a high-impact journal, we would think twice about just, you know, blowing our cover to them” EAS 1

‘Trust’ was reported as a mediating factor in decisions to share data or resources in an informal collaborative venture and was expressed both in terms of expected conduct - that fellow scientists would abide by the ‘rules of the game’ - and through personal, experience-based knowledge of individual collaborating scientists and groups. The value of the data, in terms of its perceived likelihood of forming the basis of a publication in a prestigious journal, was also reported as a factor in deciding whether or not to share, as in the example above. Established academic scientists all reported prior experience of informal collaboration ranging from estimates of 5–6 previous collaborations to having “a dozen at any one time” (EAS 7) in progress. Interestingly, industry scientists also reported informal collaborations. Given the point made above that collaborations between industrial scientists and external agencies tended to involve formal contractual arrangements as a matter of course this might seem improbable. However, in terms of scientific collaboration, industrial scientists reported “extensive” (EIS 3) interactions with colleagues in other research units within the same company as a routine occurrence:

“[N]on-formal collaborations are actually our daily work, the way we work […] we bring in the stem cell expertise […] and we have another group within our organisation that focuses and concentrates on the disease progression” EIS 2

With regard to this type of working, established industry scientists reported similar or higher levels of informal collaboration to their academic counterparts. The major difference between academic and industry informal collaborations then lies not so much in matters of scientific practice, but that for industry scientists the demarcation between intra-institutional collaboration and inter-institutional collaboration forms a hard boundary where collaboration must switch from informal to formal terms. For academic scientists, relationships with collaborators who are either already familiar or deemed trustworthy (and scientifically interesting) can facilitate relatively informal working arrangements that cross institutional boundaries, as long as they remain within the academic sphere.

## Conclusion

The findings presented above provide an insight into the dynamics of collaboration at work in contemporary stem cell science. Research collaboration was defined as: interactions between scientists that bring two or more research groups into alignment allowing them to share resources and increase the scope of do-able scientific work. Both of these elements, pooling collective resources and expanding the research potential of the combined labour force, were identified as key drivers of collaboration. The most sought-after resources were those that were a given research group could not ordinarily access within its own pool of technologies, materials, and expertise. Collaborative working primarily followed a complementary model, with co-working between scientists and research groups with different disciplinary skill sets, such as cell culture or pharmacology being necessary to address the complexity of stem cell science. This pattern is true for both academic and industrial researchers. Collaboration between multiple research groups with different skill sets and ensembles of technology within individual companies may be less visible, and thus less studied, outside the private sector. For academic scientists being able to address research questions that are seen as more socially relevant and ‘translational’ can also increase the chances of securing funding and lead to more and/or higher-value publications. For industry scientists collaboration with academic researchers offers a way to access and generate knowledge in novel areas which can nonetheless be directed to ensure that what is researched is relevant to practical company interests.

Academic stem cell scientists were familiar with ‘formal’ hierarchical, bureaucratically managed projects, but less formal collaborations, that may not involve contracts, external funding, or hierarchical organisation were also common. Shrum et al. (2001) argued that bureaucratic structures were more important than trust in enabling collaborations to work. This may be similarly true for stem cell science but in the absence of any formal structure trust still plays an important role in allowing collaborators to start working together, regardless of how this later pans out. This could be interpreted as simply another strategy for ‘getting things done’, where the security of formal collaborations is traded off against the reduced administrative burden or informal arrangements. However, respondents also described a particular moral economy of appropriate conduct in collaboration. This included a normative obligation to ensure reciprocal benefit for others who provided access to data, material and skills, and equitable granting of credit for contributions to outputs such as publications or intellectual property claims. Many elements of these findings suggest that the prevailing dynamics of collaboration in stem cell science align with, rather than challenge, the use of public-private consortia for translational research. Certainly on the evidence presented here, promoting collaboration is likely to facilitate the sharing of materials, data and expertise between groups, and indeed across sectors, that policy makers have deemed necessary for addressing translational challenges. Just as the ‘pre-collaborative’ nature of IMI stem cell projects allows industry scientists from different companies to collaborate, so too the projects’ focus on producing tools and infrastructure may also be regarded as a precursor to the ‘real’ scientific work by academic scientists and thus an appropriate (de-risked) spaced for collaborative working between competing academics (c.f. Eriksson and Webster 2008). The additional bureaucratic requirements can be burdensome to some academics, but public-private partnerships with the presence of formal project structures, agreements on publication, and intellectual property rules can provide a safe environment for all parties to exchange resources even in the absence of prior ‘trusted’ working relationships. Established academic and industry scientists are already familiar with this type of formal project arrangements from prior academic-industry partnerships and participation in European and nationally-funded projects. Moreover, the cross-disciplinary collaborative working needed for stem cell science is common to existing arrangements in both public and private sectors.

The persistence of a moral economy that encourages reciprocal sharing of material and data outside formal institutional collaborations could in theory pose a challenge to the security required by industrial collaborators. In practice though industry collaborators manage this tension both by relying on formal contractual arrangements with public sector partners and by segregating a ‘core’ element of work related to existing business areas from an ‘exploratory’ domain of emerging but unconsolidated research interests, with academic collaborations largely restricted to the latter domain. Material, know-how and data shared with academic collaborators is rarely the company’s most financially sensitive information and a slightly lower level of secrecy is often anticipated. Scientific tasks that are more routine, but closer to a company’s key business interests are more likely to be outsourced to Contract Research Organisations where inadvertent disclosure is more easily prevented. Industry scientists managing of this boundary between data and materials relevant to core and exploratory interests might also explain the perception by some academic scientists that companies are not always as forthcoming with information as the academics might wish, although this requires further study. Although public-private consortia are likely to face the general management challenges of co-ordinating geographically dispersed institutions and occasional potential differences in academic and industry cultures, none of this poses a challenge to the overall viability of public private consortia. This analysis does, however suggest some points that warrant further attention.

Traditional licensing arrangements for ‘platform’ technologies in the life sciences often involve licensing costs and terms depending on whether users are based in the academic or commercial domains. Public-private partnerships can overcome this by paying the commercial licensing fees, but the greater challenge is likely to arise if differentially licensed-technologies are themselves incorporated into communal resources produced by public-private consortia. An example is the use of CRISPR-cas 9 gene editing technology which has different licensing arrangements for public and private end users. A resource of, for example, gene-edited stem cell lines produced by a public-private consortia could potentially have difficulty securing contractual permission to make the lines available to all users on an equal basis, something that currently underpins arrangements like the European Union Innovative Medicine Initiatives.

The potential for public-private collaborations to operate in the product development sphere has also been mooted by some commentators (French et al. 2014). In addition the second phase of the IMI, which runs from 2014 to 2020, has expanded to include the development of new therapeutic products (IMI website, N.D.). This could potentially involve academic scientists collaborating in areas of knowledge production more closely associated with industry partner’s core business interests. Given the findings presented above, this is likely to significantly increase the tension between industry scientists’ need to control flows of data and academic scientists’ commitments to collegiality and need to publish. That does not mean such collaborations are doomed to fail, but any project agreement would need to be adapted to take account of this situation. If, for example, tighter secrecy was required in sharing data, protocols, reagents etc. that in turn made academic publication more difficult, then it might be appropriate to consider incorporating some other form of compensating arrangement for academic partners in the consortium agreement. It would also be important to clearly manage expectations from all parties prior to any collaborative research commencing.

There are also issues that require further investigation, especially pertaining to the distribution of benefits within ‘big biology’ endeavours (Lezaun 2013). As collaborations in stem cell science involve scientists with different disciplinary skill sets, it would be interesting to know if any particular disciplinary group tends to be placed in a subordinate or ‘service-provision’ (Calvert 2010) role more often in industrially-focused collaborations, and if so how this would affect the value of collaboration to this group of scientists? Finally, the greater vulnerability of early career researchers has been touched upon above (and see also Hackett 2005b, Davies and Horst 2015), but as yet there is little data on the balance of opportunities and protections for academics in doctoral and postdoctoral positions taking part in large scale, cross-sectoral research projects. It may be that early experience of working with industry and the opportunity to be on group papers is beneficial to their career prospects, or there may be a greater risk of their contribution being overlooked in a very large project with many senior and established scientists. Either way further investigation is warranted to ensure the rise of translational big biology benefits all those who make it possible.

## Additional file

Additional file 1: Question sheet for StemBANCC interviews on ‘Attitudes to sharing cell lines and data’. (DOCX 16 kb)

## Abbreviations

ANR (France): Agence national de la recherche (national research agency)
CDA: Confidential disclosure agreeement
CQDM: Consortium Québécois sur la découverte du médicament (Quebec consortium for drug discovery)
CRO: Contract research organisation
EAS: Established academic scientist
EBiSC: European bank for induced pluripotent stem cells
EC: European Commission
ECAS: Early career academic scientist
ECIS: Early career industry scientist
Efpia: European federation of pharmaceutical industries and associations
EIS: Established industry scientist
EU: European Union
IMI: Innovative medicines initiative
iPSC: Induced pluripotent stem cell
MTA: Material transfer agreement
NDA: Non-disclosure agreement
StemBANCC: Stem cells for biological assays of novel drugs and predictive toxicology

## Footnotes

1 That is not to say that challenges to existing norms are the only challenges large translational consortia are likely to encounter. The organisational and management difficulties of co-ordinating geographically dispersed research teams are not insignificant (Shrum et al. 2001; Hackett 2005a). Examination of these factors, though is outwith the scope of the current paper.
