# Supplementary material for: “A good collaboration is based on unique contributions from each side”: assessing the dynamics of collaboration in stem cell science
Source: Life Sci Soc Policy. 2017 May 4;13:7. doi: 10.1186/s40504-017-0053-y (PMC5418168; doi:10.1186/s40504-017-0053-y)
Supplement: Additional file 1: — Question sheet for StemBANCC interviews on ‘Attitudes to sharing cell lines and data’. (DOCX 16 kb) [file 40504_2017_53_MOESM1_ESM.docx]

Additional file 1:

**Question sheet for StemBANCC interviews on ‘Attitudes to sharing cell lines and data’.**

Part 1 | Experience of collaboration and sharing in cell science

i) Can you tell me about your position and what you do?

ii) Does your work involve human cells?

iii) If so, what sorts of cells; primary cell cultures, immortalised cell cultures, stem cells, iPS or something else?

v) Have you previously collaborated with others outside your research group in this area?

vi) Have you been involved in previous collaborative projects?

Projects meaning collaborations having external funding, involving multiple institutions, and having a formal management structure and team, deliverables etc.

vi) If yes, roughly how many? E.g. less than 5, between 5 and 10, more than 10 etc

vii) Have any of these been IMI projects?

viii) What about less formal collaborations, for example with colleagues?

- *Prompt* – how many approximately?
- *Prompt* - Are these mostly within your own institution/company?

ix) Do you collaborate more with academic or industrial partners?

x) When you collaborate do you share cell lines, data or both?

xi) Do you share unpublished data?

- *Prompt: is this usual for you?*

xii) Is there anything about sharing materials and data in cell science that makes it different from other areas of science?

- *Prompt –Cell cultures are physical objects, also alive and respond to environment. Properties change over time unless frozen.*

Part 2 | Benefits and challenges of sharing in (stem) cell science

viii) What (for you) are the main **benefits** of sharing materials and data this field?

- *Prompt: Prestige*
- *Prompt: For academics: career advancement, opportunity to employ post-docs*
- *Prompt: For industry members: improve R&D capacity, new IP, commercial advantage*

ix) What are the main **challenges** or risks of sharing materials and data this field?

- *Prompt: being ‘scooped’*
- *Prompt: intellectual property issues*
- *Prompt: publication issues*
- *Prompt: logistical / organisational issues*
- *Existing consent applying to human tissue samples*

x) Are the benefits and challenges different when collaborating with industry or academic partners?

xi) Is StemBANCC different from other projects?

- *Prompt: If so, in what ways?*

xi) Do you see StemBANCC as long term resource?

Part 3 | Features of collaboration relevant to sharing

xi) What are the main factors that affect your decision to collaborate?

- *Prompt: does it make a difference if the research is pre-competitive?*

xii) What are the benefits and challenges of research governance for collaborative projects?

xiii) What are the benefits and challenges of having a publication policy?

(including rules for attribution)

xiv) What are the benefits and challenges of having a policy and a framework for materials and data access?

xv) Are there any examples of good project governance you would like to highlight?

Part 4 | Additional

xvi) Are there any other aspects of sharing cells and data that you feel I have overlooked and that you want to comment on?

xvii) What would you say is the next important step or steps for the stem cell field?

xvii) Are there any questions you would like to ask me?
